# Perioperative management of a bleeding jejunal tumor in a patient with erythropoietic protoporphyria: A case report and literature review

**DOI:** 10.1016/j.ijscr.2019.03.048

**Published:** 2019-04-05

**Authors:** Yuki Takemoto, Shoichiro Mukai, Tetsuya Mochizuki, Masatoshi Kochi, Hiroyuki Egi, Hideki Ohdan

**Affiliations:** Department of Gastroenterological and Transplant Surgery, Applied Life Sciences, Institute of Biomedical & Health Sciences, Hiroshima University, 1-2-3 Kasumi, Minami-ku, Hiroshima 734-8551, Japan

**Keywords:** Anesthesia, Erythropoietic protoporphyria, Hemin, Surgery

## Abstract

**Introduction:**

Erythropoietic protoporphyria (EPP) is a rare disorder caused by reduced ferrochelatase activity and shows incomplete autosomal dominant inheritance. Meticulous perioperative management can avoid characteristic complications. This report describes a case of a bleeding jejunal tumor in a patient with EPP.

**Presentation of case:**

A 49-year-old man with a history of EPP was admitted to our department with abdominal distention and severe anemia. Contrast-enhanced computed tomography revealed an abdominal tumor measuring 5 cm, originating from the small bowel wall or mesentery. Tumor resection was planned after correction of anemia. Red blood cell transfusion restored his hemoglobin to acceptable levels; however, his liver function worsened. Institution of liver support therapy achieved gradual reduction in his elevated liver enzymes; however, hyperbilirubinemia persisted. He underwent tumor resection on the 12^th^ day of hospitalization. Yellow filters were used to avoid operating room light-induced tissue injury. The tumor was located in the jejunum 30 cm from the Treitz ligament toward the anal aspect. The histopathological diagnosis was desmoid-type fibromatosis of the jejunum. Postoperatively, his hemoglobin levels were stabilized; however, his serum bilirubin level remained high. His serum bilirubin level gradually decreased following hemin injections (150 mg/day).

**Discussion:**

Reducing heme synthesis and minimizing protoporphyrin generation are important perioperatively. Additionally, preventing operating room light-induced tissue burns and selecting appropriate anesthestic agents are important during surgery.

**Conclusion:**

The institution of appropriate treatment and adequate intra- and perioperative measures can ensure safe surgery in patients with EPP even under emergency conditions.

## 1 Introduction

This work has been reported in line with the SCARE criteria [1]. Erythropoietic protoporphyria (EPP) is a rare disorder showing incomplete autosomal dominant inheritance. This condition is caused by reduced ferrochelatase (FECH) activity. FECH is the terminal enzyme involved in the heme biosynthetic pathway. Reduced levels of FECH cause overproduction and accumulation of protoporphyrin (PP) in red blood cells (RBCs) and in tissues causing various disorders and symptoms [2,3]. Cutaneous accumulation of PP causes photosensitivity. PP accumulation in the liver impairs bile formation and alters the activity of hepatic membrane-bound enzymes, damages the bile duct epithelium, and precipitates biliary ﬁbrosis [[4], [5], [6]]. The incidence of EPP in the population ranges from 1:75,000 to 1:200,000 [7]. Surgical intervention is challenging and therefore rare in patients with EPP (owing to the risk of phototoxic injury). This report describes a rare case of a bleeding jejunal tumor in a patient with EPP.

## 2 Presentation of case

**Fig. 1:**
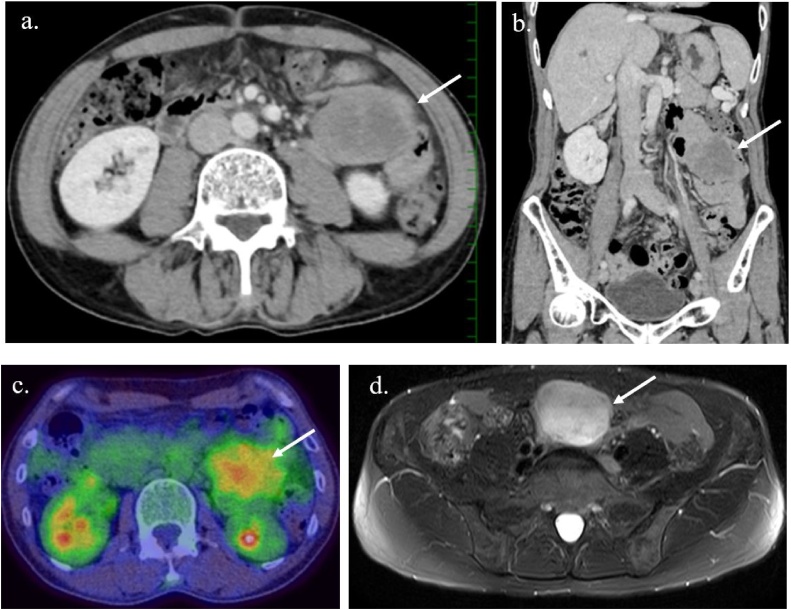
a,b: Abdominal CT indicated 5 cm, a smooth-marginated and inhomogeneous enhanced mass (white arrow). C: On PET-CT, FDG concentrated on the tumor, SUV max was 4.0. d: On MRI, the tumor showed low signal in T1-weighted images and enhanced signal in T2-weighted images. Abbreviations: CT, computed tomography; FDG, fluorodeoxryglucose; MRI, magnetic resonance imaging; PET, Positron emission tomography; SUV, standardized uptake value.

A 49-year-old man was admitted with abdominal distention to the hospital where this study was performed. He reported a several-year history of photosensitivity and EPP-induced liver dysfunction. Laboratory tests performed on admission are shown in Table 1. His Child-Pugh score showed grade A disease (6 points). Severe anemia (hemoglobin 4.2 g/dL) was diagnosed; however, he showed no worsening of liver function. Contrast-enhanced computed tomography (CT) revealed an inhomogeneous enhancing abdominal mass measuring 5 cm, with smooth margins. Positron emission tomography-CT showed a high concentration of fluorodeoxyglucose in the tumor (standardized uptake value max was 4.0). On magnetic resonance imaging (MRI), the tumor showed a low signal on T1-weighted images and an enhanced signal on T2-weighted images (Fig. 1). Gastrointestinal fiber and total colon fiber showed unremarkable findings. We concluded that the tumor originated in the small bowel wall or in the mesentry. His anemia was attributed to tumor bleeding, and tumor resection was planned after correction of his anemia.

**Table 1:** Blood examination results on patient’s admission.

WBC (/μl)	3210	T-Bil (mg/dl)	0.5	BUN (mg/dl)	14.4
RBC (×10^4^/μl)	207	D-Bil (mg/dl)	0.1	Cr (mg/dl)	0.73
Hb (g/dl)	4.2	AST (IU/l)	14		
Ht (%)	14.9	ALT (IU/l)	10	Na (mEq/l)	139
Plt (×10^4^/μl)	22.3	ALP (IU/l)	229	K (mEq/l)	3.8
Ne (%)	68	γ-GTP (IU/l)	22	Ca (mEq/l)	4.0
Ly (%)	25	LDH (IU/l)	144		
		ChE (IU/l)	134	Fe (μg/dl)	5.0
PT (%)	73	TP (g/dl)	5.9	UIBC (μg/dl)	476
PT-INR	1.17	ALB (g/dl)	3.6	RBC-protoporphyrin (MCG/DL)	308.5
		T-Chol (mg/dl)	127		

Abbreviations: Alb, albumin; AST, aspartate aminotransferase; ALT, alanine amino transferase; ALP, alkaline phosphatase; BUN, blood urea nitrogen; ChE, Cholinesterase; Cr, creatinine; D-Bil, direct bilirubin; γ-GTP, γ-glutamyl transpeptidase; Hb, hemoglobin; Ht, hematocrit; LDH, lactate dehydrogenase; Ly, lymphocytes; Ne, neutrophils; Plt, platelet count; PT, prothrombin time; PT-INR, prothrombin time-international normalized ratio; RBC, red blood cell count; T-Bil, total bilirubin; T-Chol, total cholesterol; TP, total protein; WBC, white blood cell count.

After admission, RBC transfusion restored his hemoglobin to acceptable levels; however, serum levels of total bilirubin (T-bil), aspartate aminotransferase (AST), alanine aminotransferase (ALT) and the PP content of RBCs were elevated. He received daily injections of Stronger Neo-Minophagen C (monoammonium glycyrrhizinate, glycine, aminoacetic acid, l-cysteine hydrochloride hydrate), after which AST and ALT levels gradually decreased, although T-bil remained elevated. Once again, a reduction in hemoglobin level was observed, and although liver function had not recovered adequately, tumor resection was planned to control bleeding.

On the 12^th^ day of hospitalization, he underwent tumor resection. Yellow filters were used to avoid photosensitivity secondary to operating room light. Sevoflurane, rocuronium, and fentanyl were used for general anesthesia because these agents are associated with a low risk of disease progression in patients with EPP. The tumor was located in the jejunum 30 cm from the Treitz ligament toward the anal aspect. Peritoneal dissemination and invasion of other organs were not observed. The tumor was easily removed through the umbilical incision, and partial jejunal resection and end-to-end anastomosis (Albert-Lembert method) were performed. The operation time was 104 min, and the intraoperative estimated blood loss was 55 mL.

**Fig. 2:**
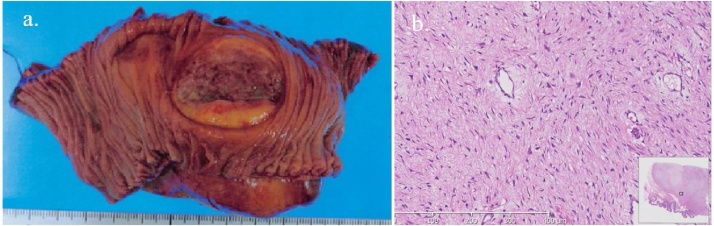
a: The tumor was located at the jejunum 30 cm anal side from Treitz ligament. b: Hyperplasia of spindle cells in the muscularis propria of the small intestine. Immunohistochemical examination of the resected specimen showed that the tumor cells stained positive for α-smooth muscle actin and Calponin and negative for CD34, S-100, c-Kit, Desmin, ALK-1, DOG1, and Caldesmon stains. The Ki-67 labeling index was 3.0%. The final histopathological diagnosis was desmoid-type fibromatosis of the jejunum.

Histopathological examination showed hyperplasia of spindle cells in the muscularis propria of the small intestine. Immunohistochemical examination of the resected specimen showed that the tumor cells stained positive for α-smooth muscle actin and Calponin and negative for CD34, S-100, c-Kit, Desmin, ALK-1, DOG1, and Caldesmon stains. The Ki-67 labeling index was 3.0%. The final histopathological diagnosis was desmoid-type fibromatosis (DF) of the jejunum (Fig. 2).

**Fig. 3:**
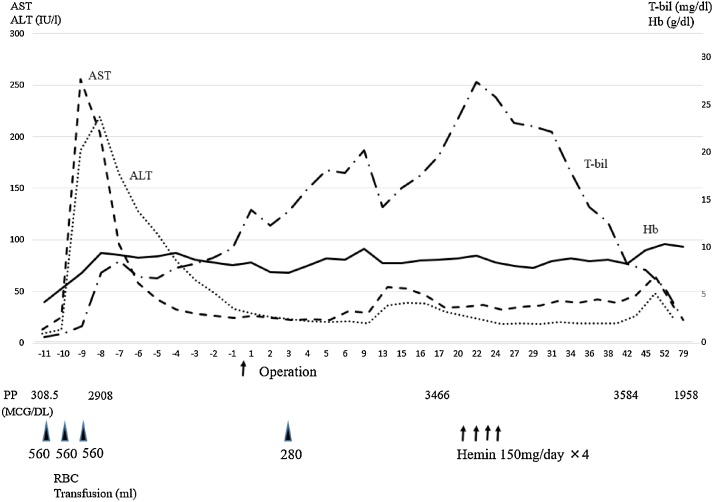
The patient's clinical course. Abbreviations: AST, aspartate aminotransferase; ALT, alanine amino transferase; T-bil, total bilirubin; Hb, hemoglobin; Ht, hematocrit; PP, protoporphyrin in red blood cells; RBC, red blood cell count.

His hemoglobin levels were stabilized postoperatively. He resumed oral intake on postoperative day (POD) 4 without digestive complications; however, his serum bilirubin levels continued to increase and hyperbilirubinemia persisted despite increased quantities of Stronger Neo-Minophagen C administered. His abdominal pain returned, and he showed elevated PP levels in RBCs. Liver supporting therapy alone could not treat this condition, necessitating injections of hemin at a dose of 150 mg/day from POD 21 to 24. After the initiation of hemin therapy, his serum bilirubin levels gradually decreased with resolution of symptoms. Hyperbilirubinemia was not observed, and he was discharged on POD 47. His clinical course is summarized in Fig. 3. Seven months have passed after surgery, no findings of recurrence were discovered, and the progression of his liver dysfunction has not been observed.

## 3 Discussion

Meticulous perioperative management is required in patients with EPP to avoid complications. The literature contains a few case reports describing surgery in patients with EPP [[8], [9], [10], [11]]: however, limited reports have described emergency surgery in these patients (as described in this case report).

The level of PP determines the degree of photosensitivity and hepatic dysfunction; therefore, maintaining low PP levels perioperatively can reduce the severity of EPP. Maintaining adequate hemoglobin levels is important because anemia triggers the activation of the heme biosynthesis pathway, thereby increasing PP production [12,13]. RBC transfusion is recommended to maintain hemoglobin levels >12 g/Dl [12,13]. This patient presented with severe anemia caused by tumor bleeding. However, preoperative liver function was inadequate, and although his hemoglobin levels subsequently improved, serum bilirubin levels continued to increase. Adequate preoperative preparation may not always be possible, particularly in emergency situations (as was noted in this present case). In this case, although transfusion improved the patient’s hemoglobin levels, persistence of tumor bleeding necessitated tumor resection despite worsened liver function. Reportedly, hemin effectively treats EPP crises [[14], [15], [16]]. In this case, the patient’s serum bilirubin levels continued to increase, and he developed abdominal pain despite stabilization of his general condition. Injection of hemin for 4 days controlled his hyperbilirubinemia. Reportedly, plasmapheresis and exchange transfusion are effective in such cases, and urodeoxycholic acid, cholestyramine, and activated charcoal are useful to enhance biliary excretion [2,6,7,15,17].

Intraoperative management of EPP primarily involves prevention of operating room light-induced tissue burns and selection of the appropriate anesthestic agents. PP absorbs light from the blue portion of the spectrum (wavelengths 380–460 nM), which triggers the formation of free radicals that precipitate tissue injury [9,18]. The usual wavelength of operating room light is 300–750 nM; thus, yellow filters, which transmit wavelengths <530 nM are used in this setting [9]. Intraoperative light-induced burns can cause cutaneous as well as abdominal organ injuries, often with serious outcomes. Third-degree skin burns around surgical scars and multiple small bowel perforations after liver transplantation have been reported [6,15]. Yellow filters used intraoperatively prevented light-induced burns in this patient. However, whether intraoperative light filters need to be used in all patients with EPP is controversial. Wahlin et al. reported that the risk of intraoperative phototoxic injury is associated with the tissue PP concentration, light intensity, and the time of exposure [11]. The risk of phototoxic injury during general surgery is lower than that associated with liver transplantation because the duration of light exposure is shorter in the former instance, and using protective filters is not always necessary [11]. In fact, a few studies have reported successful surgical procedures without using light protective measures [6,8,10]. Retrospectively, in this case, protective filters might not have been warranted because of the short operation time (104 min). However, because EPP was poorly controlled, intraoperative light protection was deemed necessary. Arranging adequate light protective measures may not always be possible, particularly in emergency situations and performing surgery using common surgical light sources could be acceptable in such patients. However, attempts should be made to shorten the operation time and control anemia.

Several anesthetic agents are associated with EPP progression. Fentanyl, droperidol, vecuronium, and isoﬂurane are associated with a low risk; therefore, fentanyl, sevoflurane, and rocuronium were used in this patient. The safety profile of propofol is controversial, which precludes its use.

Preoperative diagnosis was difficult in this case, and the lesion was considered a small bowel tumor such as a gastrointestinal stromal tumor. Surprisingly, the final diagnosis (based on histopathological confirmation) was DF. DF are rare soft tissue tumors associated with trauma, gastrointestinal surgery, pregnancy, and oral contraceptive use [19]. Patients with familial adenomatosis polyposis are at a higher risk of developing DF [20]. These risk factors were not observed in this patient; thus, the association with EPP is unclear. The recurrence rate of DF is high and strict follow-up is needed.

## 4 Conclusion

In conclusion, the institution of appropriate peri- and intraoperative measures can ensure safe surgery in patients with EPP even during emergencies.

## Consent

Written informed consent was obtained from the patient for publication of this case report and any accompanying images.

## Provenance and peer review

Not commissioned, externally peer-reviewed.

## Ethical approval

Ethical approval from Hiroshima University.

## Funding

This research received no specific grant from any funding agency in the public, commercial, or not-for-profit sectors.

## Author contribution

All authors in this manuscript contributed to the interpretation of data, and drafting and writing of this manuscript. Yu-ki Takemoto is first author of this paper. Shoichiro Mukai is corresponding author of this paper. Yu-ki Takemoto, Shoichiro Mukai, Hiroyuki Egi conceived and designed the study and drafted the manuscript. Shoichiro Mukai first diagnosed. Yu-ki Takemoto, Shoichiro Mukai, Testsuya Mochiduki, Masatoshi Kochi, Hiroyuki Egi were engaged in patient’s care in our hospital including surgery. Hiroyuki Egi contributed to study concept, and review of the final manuscript and submission of the paper. All the authors read and approved the final manuscript.

## Conflict of interest statement

None of the authors have any commercial or financial involvement in connection with this study that represents or appears to represent any conflicts of interest.

## Guarantor

Shoichiro Muki.

## Research Registration Number

The manuscript does not report the result of an experimental investigation or research on human subjects.
